# Menstrual Abnormalities in Female International Students in Japan: Changes during Pre-Arrival, Difficult, and Current Periods

**DOI:** 10.3390/ejihpe13070099

**Published:** 2023-07-21

**Authors:** Yukie Matsuura, Yoko Abe, Yoshie Motoki, Nam Hoang Tran, Toshiyuki Yasui

**Affiliations:** 1 Department of Reproductive and Menopausal Medicine, Graduate School of Biomedical Sciences, Tokushima University, Tokushima 770-8503, Japan; 2 Department of Intercultural Studies, Faculty of Literature, Shikoku University, Tokushima 771-1192, Japan; 3 Center for Faculty-Wide General Education, Shikoku University, Tokushima 771-1192, Japan; 4 Research Center for Higher Education, Tokushima University, Tokushima 770-8502, Japan

**Keywords:** female international students in Japan, menstrual symptoms, menstrual abnormalities, difficult period, stress

## Abstract

The number of Japan’s international students has rapidly increased in the last decade. This study examines the relationship between menstrual abnormalities in cycle and symptoms, stress level, and lifestyle of female international students in Japan across three time periods, namely pre-arrival, difficult, and current time periods. A cross-sectional design was employed, and data were collected through a self-administered questionnaire, including the menstrual distress questionnaire (MDQ), between December 2022 and February 2023. The questionnaire was distributed to 56 female international students from two universities in Japan, and a total of 29 valid responses were collected. We found varying menstrual cycle abnormalities and severity of menstrual symptoms across three time periods, with the difficult period after arrival in Japan displaying the highest symptom severity. Higher stress levels were significantly associated with more severe menstrual symptoms. Lifestyle habits such as alcohol consumption were also linked to menstrual symptoms. The current study emphasizes the importance of providing menstrual education, support, and resources to address international students’ unique challenges in managing their menstrual health while studying abroad. By promoting awareness, education, and access to healthcare services, universities may contribute to international students’ well-being and academic success.

## 1. Introduction

Menstruation is a physiological phenomenon, and it serves as an indicator of the status of female hormones, acting as a barometer of health. On the other hand, menstrual disorders encompass abnormalities in the menstrual cycle, volume, duration, onset or cessation, and accompanying symptoms such as premenstrual syndrome (PMS) and dysmenorrhea. Lifestyle, socio-demographic, genetic, and psychological factors [1], as well as conditions such as polycystic ovary syndrome may contribute to the menstrual disturbances [2]. According to meta-analyses, the prevalence of menstrual symptoms is significant worldwide, with 47.8% for PMS [3] and 71.1% for dysmenorrhea [4]. Factors associated with the prevalence of menstrual symptoms include dietary habits, smoking [5,6], sleep duration [7], alcohol consumption [6,8], and stress [7,9,10]. It has been established that stress is also associated with amenorrhea [10]. It has been revealed that menstrual symptoms not only affect the daily lives of young women but also have an impact on their school lives, such as absences from classes [11,12]. With regards to the female medical students, a systematic review shows a high prevalence of PMS and dysmenorrhea and impairment of academic and social quality of life due to menstrual symptoms [1].

During the last decade, the number of international students has rapidly increased in Japan [13]. International students often experience significant lifestyle changes as they leave their homes and live independently in Japan. It has been shown that mothers play essential roles as confidants when it comes to menstrual symptoms [4,14], and female university students living alone tend to experience stronger symptoms [15]. It can be inferred that international students face difficulties seeking support as they lack nearby confidants in a foreign country. In fact, the number of international students utilizing gynecological consultations, particularly for issues like irregular menstruation and dysmenorrhea at Japanese universities, has been increasing [16]. 

Moreover, a survey conducted among 153 Chinese female international students revealed that 61.4% of respondents expressed a desire to obtain information and seek advice regarding women’s sexual and reproductive health, with 85.1% of them explicitly mentioning requests related to menstruation [17]. This indicates a high demand for support related to menstrual health among international students. Regarding premenstrual symptoms, a study targeting female international students in Japan reported a prevalence of PMS: no/mild PMS at 75.0%, moderate PMS at 24.0%, and a premenstrual dysphoric disorder (PMDD) prevalence of 1.0%. Menstrual pain was identified as a contributing factor to PMS [18]. Furthermore, using a stress scale to assess the daily life stress experienced by international students, it was found that those who perceived higher stress levels also exhibited more severe PMS symptoms [19]. These studies [18,19] utilized PMS/PMDD evaluation scales but did not analyze specific individual symptoms comprehensively. Given the lack of research on symptoms experienced during menstruation, it is necessary to conduct a broader evaluation of the physical, mental, and social symptoms occurring during premenstrual and menstrual phases.

Regarding the temporal changes in menstrual symptoms, a study comparing PMS in Japanese female university students at admission and 3 months later [20] reported that symptoms worsened after 3 months. A study conducted among international students living in China for 1 year compared their experiences before coming to China. Higher stress levels were associated with menstrual changes and dysmenorrhea [21]. Another study surveyed individuals who had spent 6 months in China during cultural adaptation, comparing their experiences before coming to China and during the first 6 months of their stay (the cultural adaptation period) using the Menstrual Symptoms Questionnaire (MSQ) to assess the severity of menstrual symptoms. The study found that 18.49% of participants experienced changes in menstrual regularity and symptoms within the first six months of their stay, and the MSQ scores were significantly higher during this period. The difference in MSQ scores was associated with cultural adaptation, length of stay, perceived stress, and sleep quality [22]. In the case of international students in Japan, those with higher levels of acculturative stress also exhibited higher levels of depressive symptoms, and students who had been living in Japan for 3 years had a higher risk of developing depressive symptoms than first-year students [23]. As reported above, regardless of the years spent living abroad, international students experience menstrual disorders, among other issues. 

Previous research suggests that menstrual symptoms may worsen during periods of high stress [7,9,10], and the most difficult periods may vary by individual. However, there is a lack of empirical evidence explicitly focusing on female international students in Japan. Therefore, this study aims to investigate the relationship between menstrual abnormalities in cycle and symptoms, stress level, and lifestyle factors in female international students during three critical time periods: (1) the pre-arrival period (PP) 3–6 months before coming to Japan; (2) the difficult period (DP) as the most perceived challenging period in terms of physical and mental well-being after coming to Japan; and (3) the current period (CP). 

## 2. Materials and Methods

### 2.1. Respondents

We conducted a cross-sectional study using a self-administered questionnaire, and the data collection period was from 5 December 2022 to 28 February 2023. The study targeted all 90 female international students from two campuses at two universities in Tokushima Prefecture. The targeted universities are major academic institutions the region, known for their ability to attract international students. We excluded individuals who did not receive an explanation regarding the research and did not provide consent to participate; those who were unable to respond to the questionnaire in Japanese, English, or Vietnamese; and those who were not residing in Japan during the distribution of the questionnaire or were experiencing menstrual cessation (menstrual cycles disappear for 3 consecutive months) due to pregnancy or other reasons.

### 2.2. Measurements

The questionnaire was anonymous and available in both paper and web formats. It was prepared in two versions with identical content: (a) Japanese (with furigana) and English and (b) Vietnamese. To mitigate bias between the two response formats, we implemented several measures, including providing clear instructions to all participants, ensuring consistent content across both formats, and carefully reviewing the data for any anomalous patterns or inconsistencies. The questionnaire consisted of the following sections:

#### 2.2.1. Basic Information

This part of the questionnaire includes questions on demographic factors, including prior study enrollment in Japan, current course and year of enrollment, nationality, age, age at first menstruation, marital and childbirth status, date of arrival in Japan, history of menstrual education, and present influence on academic performance.

#### 2.2.2. Three Time Periods

This part of the questionnaire includes questions during the three critical time periods: (1) the pre-arrival period (PP); (2) the most difficult period (DP); and (3) the current period (CP). The respondent was asked to specify the time of arrival in Japan and the time of DP, and the following items were queried:

- Menstrual Status (12 items): Menstrual cycle length, regularity, duration, perceived amount of bleeding, medical history, individuals consulted regarding menstruation, level of knowledge;
- Symptoms during premenstrual and menstrual phases (49 items) using a 46-item Menstrual Distress Questionnaire (MDQ) [24,25] grouped into eight scales, including three somatic scales for pain, water retention, and autonomic reactions; three scales for negative affect, impaired concentration, and behavior change; and two scales for arousal and control. MDQ has been a standard method widely used for measuring menstruation-related symptoms. Respondents were asked to rate each item on a scale of 0 (not at all) to 4 (severely felt). The higher MDQ total scores indicate higher severity of premenstrual and menstrual symptoms. In addition, there are three items related to eating habits comprising appetite increase and wanting to eat sweets and snacks, which we found specific changes before and during menstruation [26]. Respondents were also asked to rate each item on a scale of 0 (not at all) to 4 (severely felt);
- Lifestyle (7 items): Frequency of breakfast consumption (every day, 5–6 days a week, 3–4 days a week, 1–2 days a week, never), sleep duration (<4 h, 5 h, 6 h, 7 h, 8 h, or more), exercise frequency (every day, 5–6 days a week, 3–4 days a week, 1–2 days a week, none), alcohol consumption (once a week or more, occasionally, never), smoking (yes, no), living status (living alone, living with family, living with friends, dormitory);
- Stress (10 items): overall, menstrual-related, health-related, academic-related, financial situation, interpersonal relationships, food, culture shock, homesickness, and language barrier. Respondents were asked to rate each item on a scale of 0 (not at all) to 4 (severely felt).

### 2.3. Data Collection 

Research cooperation was obtained from each university. Researchers gave face-to-face explanations to the international students using explanatory documents, and questionnaires were distributed to the international students in their preferred language. The explanatory documents included a URL and QR code for the web survey, allowing respondents to choose between paper-based or web-based responses. For paper-based responses, the participants indicated their consent by checking a consent confirmation section on the questionnaire and then answering the questions. The collected questionnaires were retrieved using collection boxes or similar methods. For the web survey, participants accessed the Survey Monkey site (https://jp.surveymonkey.com/ accessed on 5 December 2022) and checked the “consent” checkbox on the first page before proceeding with the questionnaire. It took about 20 min to complete the questionnaire. The explanatory documents were prepared in Japanese, English, and Vietnamese, and the participants were informed that participation was voluntary and that refusing to participate would not affect their academic achievements. The research was approved by the Tokushima University Hospital Research Ethics Committee for Life Sciences and Medicine (No. 4253), and permission for implementation was obtained from the respective institution’s authorities.

### 2.4. Statistical Analysis

Statistical tests were conducted using SPSS Statistics version 28.0 for Windows (IBM Corp., Armonk, NY, USA). Descriptive statistics were performed on the obtained data for each variable. Regarding lifestyle, stress, menstrual conditions (cycle, regularity, duration, amount of bleeding, gynecological visits, hormone medication usage), and individuals consulted regarding menstruation, the following were analyzed at each of the three time periods. For variables with corresponding data, Cochran’s Q test was used to compare proportions for knowledge levels of normal and abnormal menstrual conditions, knowledge levels of menstrual disorders, and PMS. Friedman’s test was used for MDQ scores, and Friedman’s or Wilcoxon signed-rank test was employed for stress fluctuations. If statistically significant differences were found, the Bonferroni test was conducted. Mann–Whitney U test and Kruskal–Wallis test were performed for variables without corresponding data, and the Bonferroni test was used in case of significant differences. Fisher–Freeman–Halton exact probability test was employed for nominal scales. For the binomial analysis, we recorded the following lifestyle categories as follows: Daily breakfast consumption (Yes/No); Sleep duration less than seven hours (Yes/No); Daily exercise frequency (Yes/No); Alcohol consumption (Yes/No); Smoking (Yes/No); Living alone (Yes/No).

## 3. Results

The questionnaire was distributed to 56 individuals, who could be mobilized during the time, and 44 responses were collected, with 25 respondents completing the survey online and 19 on paper. In order to minimize potential bias in the results, we excluded seven women who had ever used hormonal medication for contraception or gynecological treatment at any of the three time periods, as the medications may influence hormone levels in the body, leading to disruptions in menstrual regularity and menstrual pain. After excluding another eight participants who did not complete the MDQ questionnaire, the total number of valid responses was 29.

### 3.1. Respondents’ Characteristics

Five respondents were on government scholarships, twenty-three on private scholarships, and one on an exchange program. Of these, 14 were from junior colleges, 3 from undergraduate programs, 1 from a master’s program, and 11 from doctoral programs. Regarding nationality, there were 15 from Vietnam, 5 from Mongolia, 3 from Indonesia, 2 from China, and 4 from other Asian countries. The mean (±standard deviation: SD) age was 27.3 (±5.2) years, with a mean age of 23.9 (±2.4) years for individuals from Vietnam and 31.0 (±5.0) years for individuals from other countries. When considering program level, the mean age was 24.1 (±2.6) years for junior college students, 26.0 (±3.5) years for undergraduate students, and 31.5 (±5.1) years for graduate students. The mean length of stay in Japan was 36.6 (±18.2) months, ranging from 7 months to 5 years and 9 months. The mean length of stay for individuals from Vietnam was 44.3 (±10.3) months, while for individuals from other countries, it was 28.4 (±21.5) months. When considering program level, the mean length of stay was 44.2 (±11.7) months for junior college students, 41.3 (±27.1) months for undergraduate students, and 26.6 (±19.0) months for graduate students. The most challenging mental and physical health period occurred from the beginning of their stay in Japan until 4 years and 2 months later, with an average duration of 15.1 (±14.9) months since their arrival. For individuals from Vietnam, the average duration was 18.8 (±13.2) months, while for individuals from other countries, it was 11.3 (±16.1) months. When considering program level, the average duration was 18.9 (±14.4) months for junior college students, 22.7 (±22.0) months for undergraduate students, and 8.9 (±12.5) months for graduate students.

### 3.2. Background Factors Related to Menstruation

The mean (±SD) age of first menstruation was 13.4 (±2.2) years. Table 1 shows the respondents’ menstrual status and MDQ in the pre-arrival period, difficult period, and current period in relation to background factors. There was a significant change (*p* = 0.013) in the percentage of students with regular menstruation among the three time periods. There was also a significant change in the perception of normal menstrual flow (*p* = 0.022).

Total scores on the MDQ in premenstrual and menstrual distress were highest during difficult times, but there was no significant difference among the three time periods. Regarding menstrual education, 69.0% of the respondents received it at school, while 65.5% received it at home before coming to Japan. 86.2% of the respondents were aware of dysmenorrhea to some extent, and 51.7% were aware of PMS before coming to Japan. Knowledge about dysmenorrhea increased after coming to Japan. There was a significant change in the level of knowledge about PMS during the three time periods, with more people having knowledge during difficult times or at present compared to before coming to Japan. The person most commonly consulted about menstruation was the mother. When staying in Japan, fellow international students seem to play a consultant role on par with the mother regarding menstruation issues.

### 3.3. Pre- and Intra-Menstrual Symptoms

**Figure 1:**
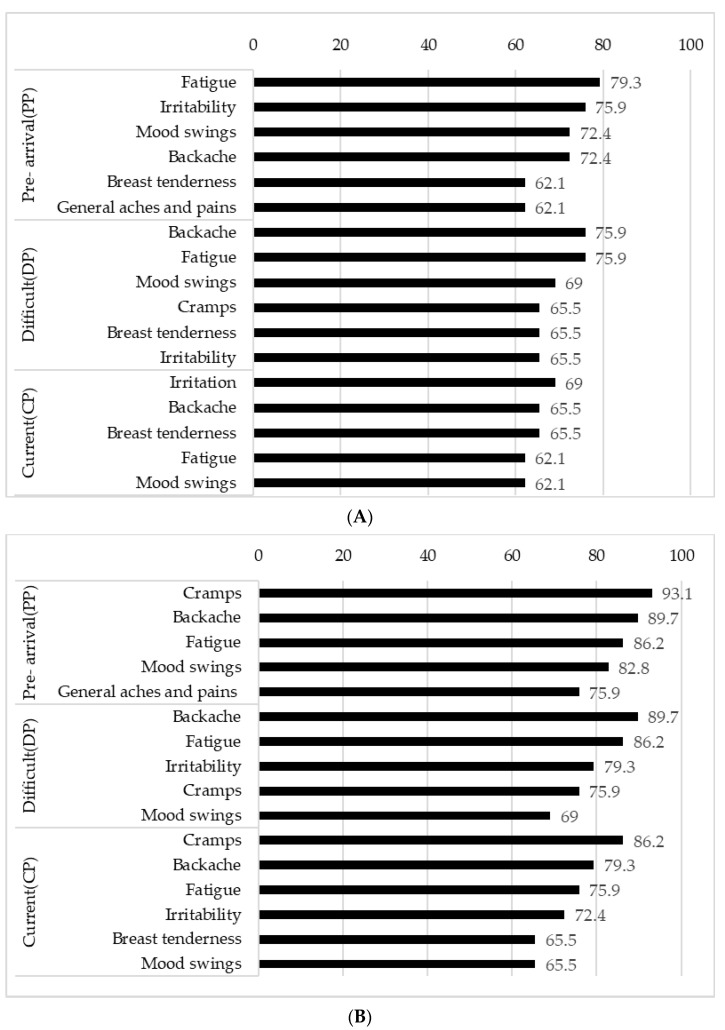
(**A**) Prevalence (%) of common MDQ symptoms at premenstrual phase by three time periods. (**B**) Prevalence (%) of common MDQ symptoms at intra-menstrual phase by three time periods.

Common premenstrual symptoms include fatigue, irritability, mood swings, and backache before coming to Japan; backache and fatigue and mood swings during difficult times; and irritability and backache or painful or tender breasts (breast tenderness) at the current period (Figure 1A). Common symptoms during the intra-menstrual phase include cramps, backache, and fatigue before coming to Japan; backache, fatigue, and irritability during difficult times; and cramps, backache, and fatigue during the current period (Figure 1B). 

During the premenstrual and menstrual phases, there was a strong desire for snack foods during three time periods (Table 2).

Regarding the impact of premenstrual symptoms on academic performance, during the current period, a considerable impact was reported by 2 respondents (6.9%) and a slight impact by 13 respondents (44.8%). Regarding symptoms during the menstrual phase, it had a considerable impact on 5 respondents (17.2%) and a slight impact on 17 respondents (58.6%). The reasons for not seeking medical consultation despite the impact of symptoms (multiple answers) were being busy for 10 respondents (34.5%), economic reasons for 6 respondents (20.7%), language issues or embarrassment for 5 respondents each (17.2%), and not knowing where to seek consultation for 1 respondent (3.4%).

### 3.4. Stress Level in Three Time Periods

**Figure 2:**
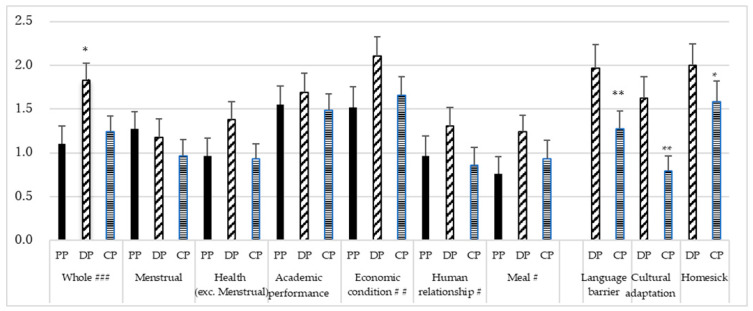
Mean and standard error of stress levels in time periods by stress type. PP: pre-arrival; DP: difficult period; CP: current period. Friedman or Wilcoxon signed test, then Bonferroni tests using median for items with significant differences. ### *p* < 0.001, ## *p* < 0.01, # *p* < 0.05 among the three groups by Friedman test. Whole, * *p* < 0.05 vs. PP and CP by Bonferroni test. * *p* < 0.05, ** *p* < 0.01.

Figure 2 shows the mean stress level in three time periods breakdown by type of stress. There were significant differences in overall stress levels among the three time periods (*p* < 0.001), with significant differences between before coming to Japan and the difficult period (*p* = 0.014) and with significant differences between the difficult and current periods (*p* = 0.038). There were also significant differences in stress levels related to the economic situation (*p* = 0.002) and interpersonal relationships (*p* = 0.024). Levels of language barrier stress (*p* = 0.001), cultural adaptation stress (*p* = 0.001), and homesickness stress (*p* = 0.038) after coming to Japan in the current period were significantly lower than those in the difficult period.

### 3.5. MDQ Total Score Change Patterns and Associated Factors in Three Time Periods

**Figure 3:**
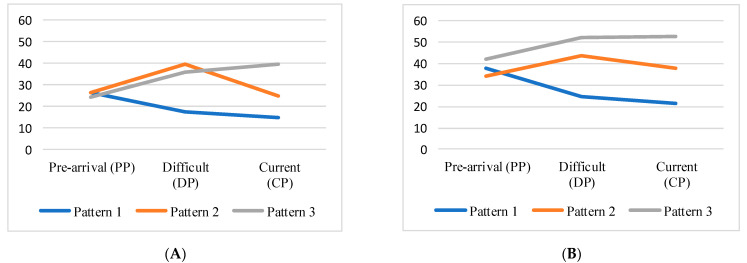
(**A**) Premenstrual change patterns by mean of MDQ total score. (**B**) Intra-menstrual change patterns by mean of MDQ total score.

When comparing the time periods with high total MDQ scores, three patterns emerged: the pattern with the highest scores before coming to Japan (pattern 1), the pattern with the highest scores during the difficult period (pattern 2), and the pattern with the highest scores at the current period (pattern 3). The means of MDQ total scores for the three patterns are shown in Figure 3A for the premenstrual phase and Figure 3B for the intra-menstrual phase. 

There were associations of changes in total MDQ score patterns with the current enrollment program and nationality. Respondents in junior colleges and undergraduate programs tended to have higher MDQ scores before coming to Japan. However, graduate program students tended to have higher MDQ scores during the difficult period. In addition, Vietnamese respondents tended to have higher scores before coming to Japan (Table 3).

### 3.6. Association between Menstrual Abnormalities and MDQ Score at Three Time Periods with Lifestyle Habits and Stress

There were no smokers. Regarding menstrual abnormalities, during the difficult period, there was a significant association between the regularity of menstruation and the severity of cultural adaptation stress (*p* = 0.002). Individuals with irregular menstruation also experienced higher levels of cultural adaptation stress. In the current period, individuals who slept <7 h had a higher prevalence of abnormal menstrual bleeding (*p* = 0.033), and those who experienced language-related stress or homesickness-related stress had a higher prevalence of abnormal menstrual cycle length (*p* = 0.024 and *p* = 0.019, respectively) (data not shown).

As shown in Table 4, during the premenstrual phase, there were associations of MDQ severity with overall, academic, and economic stress before coming to Japan. During the difficult period, MDQ intensity was associated with alcohol consumption, overall, menstrual, interpersonal, food, and cultural stress. In the current period, there were associations of MDQ intensity with alcohol consumption, overall stress, academic stress, and homesick stress. 

MDQ intensity was associated with menstrual, academic, and economic stress before coming to Japan (Table 5). During the difficult period, there were associations of MDQ intensity with overall, academic, economic, interpersonal, and cultural stress. In the current period, there were associations of MDQ intensity with alcohol consumption and overall, menstrual, academic, and interpersonal stress. 

## 4. Discussion

This study is the first attempt to investigate the changes in menstruation and accompanying symptoms among female international students before and after their arrival in Japan. Previous studies have focused on specific periods, such as the current or the cultural adaptation phases, when examining menstrual patterns. The present study examined the menstrual cycle and menstruation-related symptoms during three time periods: the pre-arrival period, the difficult period (a mean of 15.1 months after arrival in Japan), and the current period. We confirmed that international students who experienced difficulties during a specific period were more likely to report menstrual abnormalities. In addition, we revealed associations of stressors specific to international students, such as language and cultural stress, with the menstrual cycle length and regularity. Our results are consistent with some previous studies, which have found stress caused disturbances in the menstrual cycle at the hormonal level [27] and is associated with menstrual abnormalities among female university students [28]. 

There were no significant differences in the MDQ scores during the three time periods. Nevertheless, the severity of menstrual symptoms was associated with overall stress during the difficult period after arrival. In the premenstrual phase, the severity of symptoms was related to menstrual, interpersonal, food, and cultural stress. The severity of symptoms during the menstrual phase was associated with academic, economic, interpersonal, and cultural stress. In the current phase, the severity of menstrual symptoms was related to overall, menstrual, academic, and homesick stress. Previous studies have reported that menstrual symptoms were associated with daily life stress [19] and perceived stress [21] in international students. Also, changes in menstruation concerning the level of cultural adaptation during the first six months of residence have been reported [22]. Consistent with these findings, we found a significant association between stress related to cultural adaptation and MDQ scores during the premenstrual and menstrual phases, specifically during the difficult period. In addition, this study revealed that during the difficult period and in the current phase, stressors such as food and homesickness associated with the challenges of living in a foreign country were related to the severity of symptoms. These stressors were found to be associated with symptoms regardless of the duration of the stay. In a study involving female international students residing in China for one year, language barriers (25.9%), diet (20.5%), and feelings of loneliness (17.9%) were reported as sources of stress [21]. Similarly, in this survey, approximately 80% of students experienced stress related to economic conditions and homesickness during the difficult period and the current phase, highlighting the financial difficulties faced by students living in Japan.

In this study, it became evident that sleep duration is associated with the amount of bleeding during menstruation. No significant correlations existed between menstrual symptoms’ severity and lifestyle factors such as diet, sleep, and exercise. However, among students who consumed alcohol after coming to Japan, the symptoms were significantly higher during the difficult premenstrual and current menstrual phases. The association between alcohol consumption and menstrual symptoms was consistent with previous studies [6,8]. Students may drink alcohol to relieve the menstrual pain. It is necessary to explain the importance of adequate sleep and abstinence from alcohol to international students.

We found that there are three patterns of the changes in MDQ scores: higher scores during the pre-arrival period (pattern 1), higher scores during the difficult period (pattern 2), and higher scores during the current period (pattern 3). The total ratio of individuals with either pattern 2 or pattern 3 was 60.7% for premenstrual symptoms and 62.1% for menstrual symptoms, which indicates that more than half experienced a worsening of menstrual-related symptoms after coming to Japan. These results are consistent with previous studies which have reported that among Chinese international students, the proportion of individuals who reported changes in menstruation was 18.5% after staying for half a year [22] and 49% after one year [21]. The longer the stay, the more likely changes occurred compared with before coming to Japan. However, the difficult period after coming to Japan varied from immediately after arrival to 4 years for individuals experiencing a difficult period or currently having higher symptoms. Therefore, the worsening of menstruation and menstrual symptoms cannot be solely attributed to the number of months of stay. 

In addition, individuals with stronger symptoms before coming to Japan were mostly short-term college or undergraduate students. However, those with stronger symptoms during the difficult period were predominantly graduate students. This difference can be attributed to the lower average age of short-term college and undergraduate students compared with graduate students and differences in knowledge and experience regarding menstruation, menstrual symptoms, and their management. Similarly, the stronger symptoms before coming to Japan among Vietnamese individuals can be explained by their younger average age and longer duration of stay. Universities should offer menstrual education to international students upon admission and consistently after that, regardless of their duration of stay in Japan. This will enhance students’ awareness and knowledge of the subject.

There were more pain symptoms in the most common symptoms experienced before and during menstruation, according to the MDQ, followed by negative emotions. In the present study, among the symptoms experienced before menstruation, many international students reported experiencing backache, while only about half of them experienced such pain in a previous study targeting Japanese individuals [29,30]. More skin-related issues have been reported in Japanese individuals [29,30], but international students reported fewer cases in the present study. In addition, the ranking of common symptoms, such as mood swings, irritability, and fatigue, was similar to the results in Japan [29,30]. On the other hand, among the symptoms experienced during menstruation, the results in the present study were similar to those targeting Japanese university students [29]. The differences in symptoms, such as back pain before menstruation and skin-related issues during menstruation, may be attributed to the fact that the Japanese survey primarily targeted younger undergraduate students. Compared to the results of our study, which targeted mainly students from Asian countries, a study conducted in Greece has demonstrated much lower prevalence of intra-menstrual symptoms such as pain and swollen breasts, which may imply factors such as country and nationality also could influence the menstrual variation [31]. In terms of changes related to food, studies have shown that sweet foods might have been more liked and therefore selected before salty ones [32] and that women ate nearly twice as much sweet food and blander food under stress [33]. In another study conducted with Japanese participants [15,26], it was found that cravings were higher during the premenstrual phase, with many students desiring sweet foods rather than snacks. However, in the current study, the intra-menstrual phase showed a higher appetite and cravings for sweet and snack foods, with many students particularly desiring snacks. This difference may be attributed to racial or cultural variations in the study population. 

Our results are consistent with the previous literature [1,34], which has confirmed that stress, lifestyle, and socio-demographic factors can impact menstrual changes, thus influencing the well-being and mental health of international students. The challenges of adapting to a new academic environment, cultural differences, language barriers, and being away from familiar support systems can all contribute to heightened stress levels and menstrual changes.

Regardless of the duration of stay in Japan, inappropriate lifestyle habits such as lack of sleep and alcohol consumption, as well as increased stress levels, are related to the regularity of menstruation, the amount of bleeding, and the severity of accompanying menstrual symptoms. Various factors, including the academic program and nationality, influence these abnormalities. The awareness of PMS is low upon arrival in Japan, highlighting the importance of incorporating knowledge about menstruation, menstrual irregularities, appropriate lifestyle habits, and stress management during orientation sessions. Providing information that fosters understanding and connects to appropriate healthcare-seeking behaviors is necessary. In addition, establishing on-campus consultation services for menstruation concerns is crucial.

The current study is the first study conducted in Japan targeting menstrual changes among international students. The identification of three distinct patterns of menstrual changes offers valuable insights for further investigation into the factors influencing these patterns, particularly pattern 2 and pattern 3. Additionally, an intriguing finding from the study is that the perceived most difficult time period does not always align with the highest intensity on the MDQ, suggesting potential associations with age and study program. The diverse stressors experienced by international students, along with their native culture, likely contribute to the complexity of these findings.

The study has limitations that need to be considered. First, being a cross-sectional study, it does not establish cause-and-effect relationships. Secondly, recall bias is possible as respondents rely on their memory to recollect past events. The longer the duration since the events occurred, the greater the potential for bias. The study’s narrow focus on university students from two sites also led to a small sample size. Furthermore, most participants were from Asia, making applying the findings to a wider population difficult. Within the framework of this paper, we did not specifically focus on analyzing the impact of age as well as potential influence of cultural differences and overall wellness prior to coming to Japan on menstrual issues. Future research should involve longitudinal studies that include more dimensions and diverse international students, allowing for more robust and comprehensive insights.

## 5. Conclusions

This study sheds light on the experiences of international students with menstrual symptoms and their impact on daily life. It emphasizes the importance of providing menstrual education, support, and resources to address international students’ unique challenges in managing their menstrual health while studying abroad. By promoting awareness, education, and access to healthcare services, universities may contribute to international students’ well-being and academic success.

## Figures and Tables

**Table 1:** Menstrual status in the pre-arrival period (PP), difficult period (DP), and current period (CP).

		Pre-Arrival (PP)		Difficult (DP)		Current (CP)		*p*-Value ^a^
		n	%	n	%	n	%	
Cycle length	24 days or less	4	13.8	4	13.8	5	17.2	0.072
	25–38 days	20	69.0	15	51.7	19	65.5	
	39 days or more	5	17.2	8	27.6	4	13.8	
	3 months or more	0	0.0	2	6.9	1	3.4	
Regularity	Regular	16	55.2	8	27.6	12	41.4	0.013
	Somewhat irregular	7	24.1	14	48.3	11	37.9	PP-DP **
	Irregular	6	20.7	7	24.1	6	20.7	
Menstrual duration	2 days or less	0	0.0	3	10.3	2	6.9	0.066
	3–7 days	28	96.6	23	79.3	25	86.2	
	8 days or more	1	3.4	3	10.3	2	6.9	
Menstrual volume	Little	1	3.4	5	17.2	5	17.2	0.022
	Moderate	24	82.8	19	65.5	21	72.4	PP-DP *
	Plenty	4	13.8	5	17.2	3	10.3	
MDQ total score ^b^	Pre-menstrual	22.0	(11.5, 38.0)	28.0	(12.0, 43.0)	21.0	(9.0, 35.5)	0.505 ^c^
	Intra-menstrual	31.0	(15.5, 53.0)	32.0	(18.0, 55.0)	25.0	(14.6, 46.0)	0.087 ^c^
Menstruation consultant (multiple answers)	Mother	17	58.6	10	34.5	11	37.9	
	Sister	8	27.6	4	13.8	6	20.7	
	Friend	14	48.3					
	Japanese friend			1	3.4	1	3.4	
	Int’l student friend			10	34.5	9	31.0	
	School health center	1	3.4	0		0		
Did you know dysmenorrhea?	Knew	18	62.1	22	75.9	24	82.8	0.565
	Knew a little	7	24.1	1	3.4	1	3.4	
	Didn’t know much	3	10.3	6	20.7	3	10.3	
	Didn’t know	1	3.4	0	0.0	1	3.4	
Did you know PMS?	Knew	11	37.9	17	58.6	19	65.5	0.015 PP-CP *
	Knew a little	4	13.8	2	6.9	1	3.4	
	Didn’t know much	9	31.0	6	20.7	5	17.2	
	Didn’t know	5	17.2	4	13.8	4	13.8	

^a^ Cochran’s Q test, if significant, then the Bonferroni test. Analysis by normal (25–38 days) and abnormal (others) cycle length, normal (regular) and abnormal (others) regularity, normal (3–7 days) and abnormal (others) duration, normal (moderate) and abnormal (others) menstrual volume, and knowing (knew, knew a little) and not knowing (didn’t know much, didn’t know). The *p*-value of the Bonferroni test was set at * *p* < 0.05 and ** *p* < 0.01. ^b^ Median (25th percentile, 75th percentile). ^c^ Friedman test.

**Table 2:** Prevalence of appetite symptoms in three time periods.

Symptoms		Pre-Arrival (PP)		Difficult (DP)		Current (CP)	
		*n*	%	*n*	%	*n*	%
Premenstrual	Appetite increases	17	58.6	13	44.8	17	58.6
	Want to eat sweets	16	55.2	14	48.3	14	48.3
	Want to eat snacks	21	72.4	18	62.1	17	58.6
Intra-menstrual	Appetite increases	19	65.5	17	58.6	19	65.5
	Want to eat sweets	18	62.1	19	65.5	19	65.5
	Want to eat snacks	22	75.9	22	75.9	23	79.3

**Table 3:** Relationship between MDQ change pattern groups and attributes.

	Premenstrual Change N = 28							Intra-Menstrual Change N = 29						
	Pattern 1		Pattern 2		Pattern 3		*p*	Pattern 1		Pattern 2		Pattern 3		*p*
	*n*	%	*n*	%	*n*	%		*n*	%	*n*	%	*n*	%	
Number of people	11	39.3	11	39.3	6	21.4		11	37.9	14	48.3	4	13.8	
Months in Japan ^c^	40.5	(17.2)	31.6	(19.7)	38.3	(20.0)	0.601 ^a^	45.0	(15.5)	30.8	(18.4)	35.5	(22.8)	0.198 ^a^
Enrollment														
junior college	8	61.5	4	30.8	1	7.7	0.019 ^b^	9	64.3	3	21.4	2	14.3	0.001 ^b^
undergraduate	2	66.7	0	0.0	1	33.3		2	66.7	1	33.3	0	0.0	
graduate	1	8.3	7	58.3	4	33.3		0	0.0	10	83.3	2	16.7	
Nationality														
Vietnam	9	64.3	3	21.4	2	14.3	0.046 ^b^	9	60.0	4	26.7	2	13.3	0.025 ^b^
others	2	14.3	8	57.1	4	28.6		2	14.3	10	71.4	2	14.3	

^a^ Kruskal–Wallis or ^b^ Fisher–Freeman–Halton exact test was used. ^c^ Average months stayed in Japan: mean (SD).

**Table 4:** Relationship between MDQ intensity of premenstrual phase and lifestyle/stress at each period.

		Pre-Arrival					Difficult					Current				
		*n*	Mdn	25%	75%	*p*	*n*	Mdn	25%	75%	*p*	*n*	Mdn	25%	75%	*p*
Lifestyle																
Breakfast daily	Yes	17	19.0	11.0	38.5	0.845	8	19.0	2.0	30.3	0.093	10	17.5	6.8	28.0	0.286
	No	12	22.5	15.5	37.8		21	30.0	14.5	54.5		19	23.0	11.0	41.0	
Sleep > 7 h	No	9	24.0	17.5	40.5	0.216	19	28.0	13.0	41.0	0.604	20	23.5	12.8	34.3	0.167
	Yes	20	20.0	8.0	35.8		10	24.5	0.0	52.8		9	9.0	0.0	41.5	
Exercise	Yes	18	22.5	10.0	35.3	0.674	17	28.0	11.0	43.0	0.777	18	23.5	9.0	36.5	0.642
	No	11	19.0	15.0	40.0		12	27.5	14.0	48.3		11	18.0	6.0	36.0	
Alcohol	Yes	11	23.0	7.0	32.0	0.912	12	40.0	17.8	60.8	0.043	13	32.0	17.5	48.0	0.007
	No	18	18.0	11.8	40.0		17	19.0	8.5	30.5		16	16.0	1.8	26.3	
Stress																
Overall	No	11	15.0	6.0	32.0	0.044	4	12.5	3.0	16.0	0.017	7	6.0	0.0	15.0	0.003
	Mild	14	22.5	16.5	43.0		18	27.5	9.5	39.5	a–c *	20	26.0	13.3	35.8	a–b *
	Strong	4	35.5	21.3	64.0		7	63.0	28.0	67.0		2	61.0	51.0		a–c **
Menstrual	No	9	11.0	4.0	31.0	0.094	11	12.0	5.0	17.0	0.047	12	9.0	1.8	21.0	0.005
	Mild	17	22.0	16.0	37.5		12	30.0	23.8	40.8		14	33.5	19.5	43.0	a–b **
	Strong	3	36.0	31.0			6	42.0	25.0	64.8		3	29.0	12.0		
Health	No	14	14.5	6.8	26.3	0.065	8	12.5	3.0	34.8	0.097	12	16.0	6.8	23.8	0.102
	Mild	12	23.0	17.3	40.8		15	29.0	10.0	39.0		16	28.0	9.8	39.8	
	Strong	3	35.0	31.0			6	42.5	27.5	54.3		1				
Academic	No	6	6.5	1.5	21.8	0.014	6	9.0	0.0	24.8	0.109	6	3.5	0.0	14.8	0.023
	Mild	17	22.0	13.5	37.5	a–c *	17	30.0	18.0	43.0		18	23.5	16.5	35.3	a–c *
	Strong	6	35.5	22.5	54.8		6	32.5	11.5	64.8		5	46.0	11.5	61.0	
Economic	No	7	17.0	2.0	22.0	0.012	3	13.0	0.0		0.123	6	17.0	0.0	29.0	0.286
	Mild	14	18.5	11.8	32.8	a–c *	16	21.0	12.0	38.3		15	20.0	9.0	29.0	
	Strong	8	44.5	25.8	52.5		10	45.0	22.0	63.3		8	35.5	11.3	45.0	
Relationship	No	15	18.0	11.0	22.0	0.085	9	12.0	2.5	22.5	0.002	14	13.5	3.3	34.3	0.297
	Mild	11	24.0	11.0	41.0		14	29.5	16.8	40.3	a–c **	12	23.5	15.5	34.3	
	Strong	3	40.0	31.0			6	63.5	41.3	67.3		3	35.0	11.0		
Food	No	17	18.0	7.0	29.0	0.250	10	14.5	6.0	29.3	0.044	14	16.0	8.3	33.0	0.402
	Mild	11	32.0	12.0	49.0		17	31.0	15.5	43.0	a–b *	12	25.5	8.0	38.0	
	Strong	1					2	57.5	51.0			3	35.0	12.0		
Language	No						6	10.5	0.0	27.8	0.148	9	9.0	3.0	36.5	0.202
	Mild						10	32.0	15.8	42.0		17	23.0	16.0	32.0	
	Strong						13	29.0	17.5	57.5		3	46.0	12.0		
Culture	No						9	12.0	2.5	16.5	0.022	14	10.0	3.3	37.3	0.325
	Mild						11	33.0	28.0	45.0		13	24.0	19.0	32.0	
	Strong						9	30.0	17.5	65.5		2	41.5	12.0		
Homesick	No						5	0.0	0.0	38.0	0.095	5	0.0	0.0	8.5	0.008
	Mild						13	31.0	18.0	51.5		18	26.0	17.8	35.3	a–b **
	Strong						11	29.0	10.0	40.0		6	29.0	3.3	56.0	a–c *

Mdn: median; 25%: 25th percentile; 75%: 75th percentile. Mann–Whitney U test for lifestyle habits. For stress, the Kruskal–Wallis test, then Bonferroni test for items with significant differences. The result of the Bonferroni test for stress level was shown for stress levels No (a), Mild (b), and Strong (c); * *p* < 0.05 and ** *p* < 0.01.

**Table 5:** Relationship between MDQ intensity of intra-menstrual phase and lifestyle/stress at each period.

		Pre-Arrival					Difficult					Current				
		*n*	Mdn	25%	75%	*p*	*n*	Mdn	25%	75%	*p*	*n*	Mdn	25%	75%	*p*
Lifestyle																
Breakfast daily	Yes	17	31.0	13.5	56.5	0.811	8	26.0	6.3	38.0	0.218	10	21.5	10.5	37.3	0.164
	No	12	35.5	19.5	48.3		21	38.0	19.5	57.5		19	36.0	15.0	53.0	
Sleep > 7 h	No	9	31.0	22.5	44.0	0.982	19	32.0	22.0	51.0	0.735	20	29.0	20.0	46.5	0.234
	Yes	20	37.0	13.3	56.0		10	38.5	6.5	57.8		9	14.0	2.0	47.5	
Exercise	Yes	18	33.0	14.0	51.5	0.877	17	35.0	18.0	60.5	0.556	18	29.0	16.3	46.5	0.877
	No	11	31.0	17.0	56.0		12	30.5	17.5	39.8		11	23.0	14.0	47.0	
Alcohol	Yes	11	31.0	21.0	59.0	0.877	12	44.0	27.5	62.0	0.088	13	45.0	20.0	57.0	0.022
	No	18	37.0	14.0	51.5		17	22.0	10.5	45.5		16	22.5	6.8	35.8	
Stress																
Overall	No	11	21.0	14.0	42.0	0.074	4	10.5	2.0	19.8	0.002	7	6.0	0.0	15.0	0.001
	Mild	14	30.0	17.3	51.0		18	29.5	18.5	51.8	a–c **	20	35.5	20.5	49.5	a–b **
	Strong	4	56.0	48.5	74.8		7	62.0	38.0	74.0		2	80.5	47.0		a–c **
Menstrual	No	9	17.0	11.0	38.5	0.044	11	19.0	8.0	38.0	0.051	12	14.5	4.5	24.5	0.009
	Mild	17	31.0	21.5	56.0		12	33.5	23.8	52.0		14	40.0	23.5	48.0	a–b *
	Strong	3	50.0	46.0			6	52.5	41.8	77.0		3	53.0	20.0		
Health	No	14	19.0	12.0	44.0	0.055	8	20.0	3.5	54.0	0.087	12	22.5	8.0	44.8	0.190
	Mild	12	37.5	28.3	56.0		15	29.0	19.0	51.0		16	28.5	17.8	48.3	
	Strong	3	46.0	39.0			6	47.0	38.0	71.8		1				
Academic	No	6	15.5	10.8	28.3	0.013	6	14.5	1.5	33.8	0.017	6	10.0	0.0	21.8	0.006
	Mild	17	31.0	16.5	45.5	a–c *	17	32.0	22.0	50.5	a–c *	18	29.0	19.3	41.0	a–c **
	Strong	6	56.0	42.3	86.5		6	62.5	33.5	77.0		5	62.0	33.5	104.5	
Economic	No	7	17.0	13.0	28.0	0.010	3	13.0	0.0		0.027	6	14.5	0.0	36.0	0.062
	Mild	14	30.0	13.5	45.5	a–c **	16	29.5	19.8	47.5	a–c *	15	24.0	15.0	44.0	
	Strong	8	52.5	37.8	75.0		10	52.5	32.8	65.8		8	45.5	24.0	86.8	
Relationship	No	15	22.0	14.0	42.0	0.074	9	16.0	5.0	22.0	<0.001	14	19.5	5.5	41.3	0.041
	Mild	11	39.0	21.0	59.0		14	33.5	25.8	52.3	a–b *	12	29.0	20.0	46.3	a–c *
	Strong	3	56.0	46.0			6	64.0	50.0	83.0	a–c ***	3	95.0	40.0		
Food	No	17	22.0	14.0	46.0	0.301	10	24.5	10.3	53.8	0.151	14	27.5	12.0	45.5	0.578
	Mild	11	40.0	29.0	59.0		17	35.0	19.5	53.0		12	24.5	16.0	48.3	
	Strong	1					2	82.0	54.0			3	40.0	20.0		
Language	No						6	16.0	1.5	38.8	0.073	9	17.0	3.0	53.0	0.274
	Mild						10	26.0	18.5	51.5		17	25.0	17.5	42.0	
	Strong						13	39.0	30.5	62.5		3	62.0	20.0		
Culture	No						9	16.0	5.0	24.5	0.010	14	16.0	5.5	45.5	0.310
	Mild						11	35.0	22.0	54.0	a-c **	13	33.0	22.5	47.5	
	Strong						9	51.0	33.5	64.5		2	67.0	20.0		
Homesick	No						5	8.0	1.0	43.5	0.116	5	6.0	0.0	52.0	0.130
	Mild						13	35.0	24.5	57.5		18	34.0	21.5	44.3	
	Strong						11	39.0	17.0	54.0		6	33.5	11.5	75.0	

Mdn: median; 25%: 25th percentile; 75%: 75th percentile. Mann–Whitney U test for lifestyle habits. For stress, the Kruskal–Wallis test, then Bonferroni test for items with significant differences. The result of the Bonferroni test for stress level was shown for No(a), Mild(b), and Strong(c); * *p* < 0.05, ** *p* < 0.01, and *** *p* < 0.001.

## Data Availability

Data are not publicly available due to privacy restrictions.
